# Engineered Polylactic Acid (PLA) Microcapsules for Spatiotemporally Coupled Delivery and Synergistically Enhanced Dual Immunity

**DOI:** 10.3390/pharmaceutics18040456

**Published:** 2026-04-09

**Authors:** Shaoyu Guan, Yu Zhang, Hongyi Liu, Jialu Li, Lisha Wang, Jing Wang, Hua Yue, Fenghua Xu

**Affiliations:** 1 Pharmaceutical Sciences Research Division, Department of Pharmacy, Medical Supplies Centre of PLA General Hospital, Medical School of Chinese PLA, Beijing 100853, China; guanshaoyu@301hospital.com.cn (S.G.);; 2 State Key Laboratory of Biopharmaceutical Preparation and Delivery, Institute of Process Engineering, Chinese Academy of Sciences, Beijing 100190, China; zhangyu21@ipe.ac.cn (Y.Z.); liuhongyi22@ipe.ac.cn (H.L.);; 3 University of Chinese Academy of Sciences, Beijing 100049, China

**Keywords:** PLA microcapsules, delivery system, composite adjuvant, dual immunity

## Abstract

**Background:** With the evolving paradigm of vaccine development, microcapsules have attracted considerable research interest as particulate adjuvants over the past decades. However, the rational engineering design of microcapsule-based composite adjuvant systems to elicit robust immune responses remains a significant challenge. **Methods:** This study developed polylactic acid (PLA) microcapsules with spatiotemporally coupled delivery and immunopotentiator properties. The resulting formulations were assessed for humoral and cellular immune responses in mice. **Results:** We prepared uniform-sized microcapsules (MC) and formulated them with monophosphoryl lipid A (MPLA) as a composite component (MPLA@MC), with hydrodynamic diameters of 4.58 μm and 4.12 μm, respectively. Such composite adjuvants, when loaded with ovalbumin (OVA) to form OVA@MC and OVA&MPLA@MC, promoted cellular uptake and activation, exhibiting preferred lysosomal escape advantages. For in vivo experiments, microcapsule-based vaccines elevated serum levels of IgG antibody, and OVA&MPLA@MC induced Th1-biased antibody responses. Specifically, OVA&MPLA@MC also elicited strong cellular immune responses compared to other vaccines, as evidenced by increased secretion of Interferon-γ (IFN-γ) in mouse splenocytes and Granzyme B (Gzmb) in T cells. Mechanistically, muscle tissues at the injection site showed that microcapsule-based vaccines enhanced the recruitment for phagocytosis. Meanwhile, bulk RNA sequencing (RNA-seq) confirmed extensive activation of immune responses and related signaling pathways. **Conclusions:** This rationally designed composite strategy for spatiotemporally coupled delivery serves as a potent platform for orchestrating synergistic immune responses, opening up new avenues for the development of effective therapeutic and anti-infectious vaccines.

## 1. Introduction

Although aluminum adjuvants have historically dominated the landscape in vaccine formulation, their utility is constrained by a specific immunological profile: they are effective at eliciting antibodies but fail to trigger robust cell-mediated immune (CMI) responses. This deficiency presents a significant hurdle for eliminating intracellular infections [1,2].

Monophosphoryl lipid A (MPLA), a Toll-like receptor (TLR) agonist, has been repeatedly shown to induce a predominantly Th1-biased immune response, which is advantageous for cellular immunity [3,4,5,6]. Moreover, the efficacy of MPLA hinges on rational carrier design to ensure proper release for targeted delivery. Notably, a clinically successful AS01_B_ adjuvant system (a liposome-based formulation) incorporates MPLA and QS-21 saponin as key immunostimulants [7,8]. However, the inclusion of QS-21 additionally raises concerns regarding hemolytic toxicity, and the composite of molecular adjuvants and liposomal carriers is prepared by simple physical mixing. Consequently, achieving synergistic potentiation with rational design of composite adjuvants as functional units within a safe delivery paradigm remains a critical unmet challenge in vaccinology.

Particulate formulations have now emerged as a pivotal adjuvant platform in the development of vaccine delivery systems [9,10]. Among the numerous biomaterials, FDA-approved polylactic acid (PLA) represents a promising candidate owing to its well-documented biocompatibility and biodegradability [11,12,13,14]. In terms of biodistribution, nanoparticles (<500 nm) [15] preferentially exploit lymphatic drainage to directly target draining lymph nodes [16], while larger microspheres (>10 μm) [17] tend to establish a local “antigen depot” and modulate the microenvironment [16,18]. Notably, particles within the intermediate size regime remain largely unexplored. Therefore, filling this size void holds significant promise for uncovering structure–activity relationships and functional outcomes in applications.

Building upon our established expertise in particle preparation technology [19], herein, we exploited structural engineering to construct uniform PLA microcapsules with an optimized size scale that bridged the size gap. Extending beyond this foundation, we developed a rationally integrated adjuvant platform that synergistically coupled microcapsule-based carriers with the molecular immunostimulant MPLA. Utilizing the water-in-oil-in-water (W/O/W) double emulsion solvent evaporation method, we dissolved the hydrophilic antigen (ovalbumin, OVA) in the internal aqueous phase and dissolved MPLA in the oil phase, achieving efficient coencapsulation of antigen and adjuvant. Different from single-component formulations or simple physical mixing, this integrated system achieved spatiotemporally coordinated immune activation. Specifically, surface-located MPLA acted as the initial effector, releasing upon microcapsule erosion to engage cell-surface TLRs and prime cellular recognition. Concurrently, the optimally sized microcapsules facilitated efficient internalization, delivering the encapsulated OVA for intracellular processing. Our composite strategy relied on the coordination of the cellular internalization of OVA-loaded microcapsules with the precise molecular targeting of MPLA in distinct functional compartments. It facilitated efficient intracellular delivery and cross-presentation of OVA, thereby orchestrating a balanced profile of effective dual immunity. The resulting immune responses included massive recruitment of macrophages, production of high-level IgG and Th1-biased antibodies, elevated numbers of IFN-γ-secreting splenocytes, and increased cytokine production. Consequently, these engineered PLA-based microcapsules provide a tunable platform for composite adjuvants to reinforce both humoral and cellular immune responses, warranting further exploration for potent therapeutic and anti-infectious vaccine models.

## 2. Materials and Methods

### 2.1. Mice

Female C57BL/6 mice (6–8 weeks old, weighing 19 ± 2 g) were housed at the Institute of Process Engineering (IPE) Laboratory Animal Center under a specific pathogen-free, controlled temperature environment (20 ± 1 °C; 55 ± 10% humidity) with a 12 h light/dark cycle (60–300 lx) in individually ventilated cages furnished with bedding, nesting materials and environmental enrichment items with a maximum of six mice per cage. Food and drinking water were provided at all times. Following transport, the mice underwent a one-week acclimatization period before the experiments began.

### 2.2. Materials

O-Desacy1-4-monophosphoryl lipid A was purchased from Avanti Polar Lipids (Alabaster, AL, USA). Ethyl acetate and sorbitan monooleate (Span 80) were supplied by Xi Long (Guangzhou, China). Poly(vinyl alcohol) (PVA-217) was obtained from Kuraray (Tokyo, Japan). Poly(D,L-lactic acid) (PLA, Mw~50 kDa) was sourced from Dai Gang (Jinan, China). AS01_B_, a domestic generic version, was obtained from Dumabio (Shanghai, China). OVA and Harris hematoxylin and eosin Y were sourced from Sigma Aldrich (St. Louis, MO, USA). FITC-OVA and brefeldin A were purchased from Solarbio (Beijing, China). Lipophilic Cy5 was obtained from Fanbo (Beijing, China). A Micro BCA™ Protein Assay kit and LysoTracker Red DND-99 were obtained from Thermo Fisher Scientific (Waltham, MA, USA). The culture medium for macrophages and splenocytes was DMEM and RPMI 1640 medium with 10% (*v*/*v*) fetal bovine serum (FBS), 100 units/mL penicillin, and 100 μg/mL streptomycin (Gibco, Waltham, MA, USA). Horseradish peroxide (HRP)-conjugated rabbit-anti-mouse IgG, IgG1 and IgG2c antibodies were purchased from Abcam (Cambridge, UK). All fluorochrome-labeled anti-mouse monoclonal antibodies were provided from Biolegend (San Diego, CA, USA) and BD Biosciences (San Jose, CA, USA). Ninety-six-well PVDF membrane filtration plates were obtained by Merck Millipore, and mouse IFN-γ ELISpot PLUS (ALP) was supplied by Mabtech (Nacka Strand, Sweden). All fluorochrome-labeled rabbit anti-mouse polyclonal antibodies were purchased from Servicebio (Wuhan, China). PMA and ionomycin were purchased from Dakewe (Shenzhen, China). Analytical grade was the standard for all other reagents.

### 2.3. Fabrication of Microcapsule Vaccines

PLA microcapsules were prepared using a W/O/W double emulsion solvent evaporation method following a previously reported procedure with minor modifications [18,20,21]. Briefly, 1 mL of the inner aqueous phase (W_1_) containing OVA (100 μg/mL) was emulsified into 1 mL of the oil phase (O) consisting of 100 mg of a polymer mixture (PLA as the main matrix and 0.05% (*w*/*v*) Span 80 as the emulsifier) using an ultrasonifier (BRANSON, S-450D, Danbury, CT, USA) operated at 100 W for 20 s. The prepared primary emulsion was added to the external aqueous phase (W_2_) containing 15 mL of 2% (*w*/*v*) PVA, followed by homogenization at 12,000 rpm for 120 s using a homogenizer (IKA, ULTRA T18, Staufen, Germany). The obtained emulsion was transferred to ultrapure water and stirred to evaporate the organic solvent and solidify the microcapsules. The microcapsules were collected by centrifugation, washed three times with ultrapure water, and stored as suspensions at 4 °C for subsequent in vitro and in vivo experiments. For fluorescent labeling, Cy5 was added to the oil phase; for MPLA-containing microspheres, MPLA (20 μL in ethyl acetate solution) was added to the oil phase. Other procedures remained unchanged. The final products were designated OVA@MC and OVA&MPLA@MC.

### 2.4. Physical Characteristics of Microcapsule Vaccines

Microcapsule samples were dried for 12 h and used for the observation of shape and surface morphology by field emission scanning electron microscopy (SEM) (JEOL JSM-6700F, Tokyo, Japan). To prevent sample charging, electron images were captured using a large field detector in low vacuum mode. Remaining parameters, including voltage, spot size, pressure, and working distance, were optimized individually for each sample, yielding mean values of 6.0 kV (HV), 4.5 (spot size), 100 Pa (pressure), and 8.0 mm (working distance).

The hydrodynamic diameter (Z-average size) and polydispersity index (PDI) of microcapsules were determined by dynamic light scattering (DLS) according to the manufacturer’s instructions. Microcapsules were diluted to 1 mL with 10 mM PBS and added to 1.5 mL of two-sided disposable polystyrene semimicro cuvettes (Fisherbrand, Shanghai, China). Measurements were executed in triplicate, each consisting of ten runs at 20 °C, using a Nano ZS Zetasizer with a 633 nm laser and 173° optics (Malvern Panalytical, Malvern, UK).

To evaluate encapsulation efficiency, microcapsule vaccines were centrifuged at 800× *g* for 3 min to remove unencapsulated OVA. The supernatant was collected for quantitative analysis of encapsulation efficiency, which was determined at room temperature using the Micro BCA™ Protein Assay kit and the absorbance was measured at 562 nm using a microplate reader (Tecan Infinite M200, Männedorf, Switzerland). The encapsulation efficiency was calculated by the subtraction method with the following equation:

Encapsulation efficiency% = (1 − Cs/Ct) × 100

where Cs is the content of free OVA in the supernatant and Ct is the total input content of OVA.

The zeta potential of microcapsules was determined using the Nano ZS Zetasizer. Prior to measurement, the microcapsules was diluted 10 × with ultrapure water to a total volume of 1 mL to achieve an appropriate scattering intensity. The diluted suspension was transferred to a disposable folded capillary cell (DTS1060), and the zeta potential was calculated from the electrophoretic mobility using the Smoluchowski equation. Measurements were executed in triplicate, each consisting of six runs at 20 °C.

### 2.5. Culture of Mouse Peritoneal Macrophages

After sacrificing the mice, peritoneal macrophages were isolated from peritoneal lavage fluid as described previously [22]. Cells were cultured at 37 °C/5% CO_2_ in complete DMEM (with 10% FBS, 100 units/mL penicillin, and 100 μg/mL streptomycin). To remove nonadherent cells and cellular debris, the medium was changed after 12 h of culture. Primary cultured macrophages were used for in vitro experiments and identified by flow cytometry.

### 2.6. Cellular Uptake and Activation Study

To assess the uptake of antigen and cellular activation, microcapsules loaded with Cy5-OVA and OVA without fluorescence labeling were prepared, and mouse peritoneal macrophages were cultured in 12-well plates (Corning, Corning, NY, USA) with each well containing 10^6^ cells and 1 μg/mL OVA in 1.5 mL of complete DMEM. Following coincubation with OVA and microcapsules for 12 h, with untreated cells serving as a negative control, the cells underwent three PBS washes to clear away any free OVAormicrocapsulesand were subsequently analyzed by flow cytometry.

### 2.7. Intracellular Trafficking Study

The internalization and intracellular trafficking of microcapsules were characterized by confocal laser scanning microscopy (CLSM, Leica TCS SP8, Wetzlar, Germany). Mouse peritoneal macrophages were plated onto multiwell glass-bottom dishes (Matsunami, Osaka, Japan) at a density of 10^5^ cells per dish using 400 µL of complete DMEM. After complete adherence of the cells, FITC-OVA and Cy5-labeled microcapsules were added to coincubate at 37 °C/5% CO_2_ for 6 h. LysoTracker Red DND-99 was used as an organelle marker for lysosome staining, diluted 1:10,000 in PBS, and incubated with 100 µL of dye per dish at 37 °C for 15 min. The intracellular colocalization of OVA and lysosomes was observed using CLSM. Representative confocal images were pseudocolored using ImageJ2 software (NIH, Bethesda, MD, USA) (green: OVA and red: lysosome), and color separation indicated successful lysosomal escape.

### 2.8. Immunizations

#### 2.8.1. Vaccine Formulations

OVA@MC and OVA&MPLA@MC were prepared as described above. The OVA vaccine delivered by AS01_B_ (OVA&AS01_B_) was prepared by simple mixing of OVA with the AS01_B_ adjuvant system at a concentration of 100 μg OVA/mL prior to immunization. Free OVA was dissolved in saline at the equivalent dose.

#### 2.8.2. Immunization Procedure

Mice were randomly divided into five groups (*n* = 6 per group), including the PBS-treated group as a negative control, and each mouse was considered an experimental unit. Mice were vaccinated intramuscularly in the left quadriceps as follows: (i) OVA alone, (ii) OVA&AS01_B_, (iii) OVA@MC, and (iv) OVA&MPLA@MC; they received 50 μL of vaccine formulation per injection at 14-day intervals (days 0 and 14). Mice were euthanized on day 35 postimmunization (study endpoint) for terminal sample collection. For microcapsule-based formulations, mice were immunized with a volume of 5 μL microcapsules per mouse, corresponding to an OVA dose of 5 μg per mouse. Blood and spleens were collected from mice four weeks post-final immunization. The choice of doses, frequency, and administration routes was guided by previous studies [23].

### 2.9. Enzyme-Linked Immunosorbent Assay (ELISA)

ELISA was used to determine IgG, IgG1 and IgG2c antibodies against OVA in serum as previously described [24]. After anesthesia, blood samples were collected from the retro-orbital venous plexus on days 0, 7 and 35. The serum collected on day 0 served as a negative control. The blood samples were then centrifuged at 8000× *g* for 15 min to obtain serum. Additionally, 96-well high-binding polystyrene microplates (Corning, Corning, NY, USA) were coated with 2 µg/mL OVA at 4 °C overnight in coating buffer and blocked with 5% bovine serum albumin (BSA, Solarbio, Beijing, China) in PBS at 37 °C for 2 h. Serially diluted serum was added to the ELISA plates and incubated at 37  °C for 1 h, followed by a wash (PBST: PBS/0.05% Tween-20) and the addition of 100 µL of goat anti-mouse secondary antibodies against IgG (ab97040, 1:5000), IgG1 (ab98693, 1:5000), and IgG2c (ab98722, 1:5000). After a 30 min incubation at 37 °C, the plates were washed again and added with 100 µL tetramethylbenzidine substrate (TMB; Beyotime, Hangzhou, China) per well for 15 min in the dark. After the addition of a stop solution (Solarbio, Beijing, China), the absorbance at 450 nm was recorded using a microplate reader. The endpoint titers were determined as the reciprocal of the highest sample dilution that produced an optical density at 450 nm (OD_450_) value exceeding 2.1-fold of the mean OD_450_ value from the PBS group.

### 2.10. Splenocyte Culture

At the endpoint, spleens were isolated and homogenized. Splenocytes were suspended in 48-well tissue culture plates at 5 × 10^6^ cells/mL in complete RPMI 1640 and stimulated in vitro with 4 µg/mL OVA. In addition, the splenocytes were treated with 4 µg/mL brefeldin A overnight (6–10 h). The plates were incubated at 37 °C/5% CO_2_, and the splenocytes were then harvested and stained for spectral flow cytometry the next day as described previously [22].

### 2.11. Antibody Staining and Flow Cytometry

Mouse peritoneal macrophages were harvested by trypsinization with 0.25% trypsin-EDTA (Gibco, Waltham, MA, USA) at 37 °C for 5 min. Harvested macrophages and splenocytes in tubes were stained with Zombie UV^TM^ Fixable Viability Kit (423113, 1:80) and Zombie Yellow^TM^ Fixable Viability Kit (423103, 1:80), respectively. First, after incubation with 80 µL of dye per tube at 4 °C for 30 min, the cells underwent two successive washes with staining buffer (2% FBS, 2 mM EDTA). Then, the cells were stained with monoclonal antibodies targeting various cell surface markers. For the experiment of macrophages evaluated by conventional flow cytometry, the following antibodies were used: anti-F4/80-FITC (BM8, 123107), anti-CD11b-PE/Cyanine 7 (M1/70, 101215), anti-CD86-PE (A17199A, 159204) and anti-I-A/I-E-PerCP (M5/114.15.2, 107623). For the splenocyte experiment evaluated by spectral flow cytometry, the following antibodies were used: anti-CD45-Brilliant Violet 510^TM^ (30-F11, 103137), anti-CD3-FITC (17A2, 100203), anti-CD69-Spark PLUS B550^TM^ (H1.2F3, 104561) and anti-I-A/I-E-Spark PLUS UV395 (M5, 107679). The monoclonal antibody mix was prepared and diluted 1:100 in staining buffer, followed by the addition of 15 µL of True-Stain^TM^ Multi-Fluor Buffer (Biolegend, San Diego, CA, USA). Cells were incubated with 100 µL of dye per tube at 4 °C for 30 min, washed twice with staining buffer, and then fixed with fixation buffer (Biolegend, San Diego, CA, USA) at room temperature for 20 min. After fixation, intracellular staining was performed using a mixture of diluted monoclonal antibodies (anti-Gzmb-Alexa Fluor 647, GB11, 515405; anti-IL-2-PE/Cyanine5, JES6-5H4, 503824) in Intracellular Staining Perm Wash Buffer (Biolegend, San Diego, CA, USA). Cells were incubated with 100 µL of dye per tube at room temperature for 30 min, washed twice again, and resuspended in 250 µL staining buffer.

Flow cytometry data were collected using a CytoFLEX Mosaic spectral flow cytometer (Beckman Coulter, Brea, CA, USA), and the analysis of these data was conducted using FlowJo v10.8.1 software (BD, Ashland, OR, USA) and CytExpert for Spectral v1.2.0.4 software according to the manufacturer’s instructions (Beckman Coulter). The analysis strategy is shown in Figure S2B. Additionally, t-distributed Stochastic Neighbor embedding (t-SNE) was performed using Cytobank v10.7 software (Cytobank.cn) for dimensionality reduction and visualization of high-dimensional cytometry data [25].

### 2.12. IFN-γ Enzyme-Linked Immunospot (ELIspot) Assay

The assay was executed according to the manufacturer’s instructions (Mabtech). In brief, 96-well PVDF-membrane filtration plates were pre-coated with anti-mouse IFN-γ antibody at 15 µg/mL and incubated overnight at 4 °C. At the endpoint, mouse splenocyte suspensions were collected followed by red-cell lysis and cell counts and then plated at 4 × 10^5^ per well in triplicate with 4 μg/mL OVA. After being restimulated overnight at 37 °C in a cell incubator, plates were rinsed with cold ultrapure water and then incubated with biotinylated anti-mouse IFN-γ antibody at room temperature for 2 h. Following five washes with PBST, the plates were incubated with the streptavidin-ALP conjugated antibody for 1 h. Subsequently, 100 µL of NBT/BCIP substrate (Pierce, Rockford, IL, USA) was introduced into each well for coloration of spots. Then, the plates were thoroughly washed with ample ultrapure water to stop the reaction. Once the plates were fully air-dried, an automated ELISpot reader (AT-Spot 2100, Beijing, China) was used to count the spots.

### 2.13. Histology Examination

Tissue specimens were harvested on day 3 postimmunization. The quadriceps muscle of the left thigh was removed and fixed in 4% paraformaldehyde solution (Solarbio, Beijing, China) overnight. Samples were dewaxed with Environmentally Friendly Dewaxing Transparent Liquid, subjected to gradient ethanol dehydration, and embedded in paraffin, and continuous cross-sections (3μm thick) were prepared. Tissue sections were stained with hematoxylin and eosin Y (H&E), and other consecutive serial sections were prepared for immunofluorescence (IF) staining according to standard histological protocols [26,27]. After blocking, the specimens were incubated overnight at 4 °C with the following primary polyclonal antibodies: rabbit anti-F4/80 (1:2000, GB113373) and rabbit anti-CD11c (1:2000, GB11059). Subsequently, the sections were incubated with HRP-conjugated goat anti-rabbit secondary antibody (IgG, 1:400, GB23303, Servicebio) at room temperature for 1 h. The signal was amplified using tyramide signal amplification (TSA) with IF555-tyramide (1:400, G1233, Servicebio) and IF488-tyramide (1:400, G1231, Servicebio) as described previously [28]. The images were acquired using an inverted fluorescence microscope (Olympus SWE-CX63, Tokyo, Japan) and were processed by CaseViewer v2.4 (3DHISTECH, Budapest, Hungary) and ImageJ2 software.

### 2.14. RNA Extraction and Library Construction

On day 3 postimmunization, the mice were sacrificed, and the left quadriceps muscles (vaccination site) were collected. Total RNA extraction and purification were performed with TRIzol reagent (Invitrogen, Carlsbad, CA, USA) according to the manufacturer’s guidelines. A NanoDrop ND-1000 (NanoDrop, Wilmington, DE, USA) was used to quantify the RNA amount and purity in each sample. The RNA integrity was determined using a Bioanalyzer 2100 (Agilent, Santa Clara, CA, USA), with a RIN exceeding 7.0, and confirmed by electrophoresis on a denaturing agarose gel. Poly(A) RNA was extracted from 1 μg of total RNA with Dynabeads Oligo dT25-61005 (Thermo Fisher, Waltham, CA, USA) undergoing two purification rounds. Then, the poly(A) RNA was divided into small fragments with the help of the Magnesium RNA Fragmentation Module (NEB, Ipswich, MA, USA) at 94 °C for 5 to 7 min. Next, SuperScript™ II Reverse Transcriptase (Invitrogen, Carlsbad, CA, USA) was employed to reverse-transcribe the cleaved RNA fragments into cDNA, which was subsequently used to synthesize U-labeled second-stranded DNAs with *E. coli* DNA polymerase I, RNase H (NEB, Ipswich, MA, USA) and dUTP Solution (Thermo Fisher, Waltham, MA, USA). To prepare for ligation with indexed adapters, an A-base was added to the blunt ends of each strand. These adapters featured a T-base overhang to facilitate attachment to the A-tailed fragmented DNA. Fragments were ligated with single- or dual-index adapters, followed by size selection using AMPureXP beads. Following treatment of the U-labeled double-stranded DNAs with the heat-sensitive UDG enzyme (NEB, Ipswich, MA, USA), the ligated products underwent PCR amplification under these conditions: initial denaturation at 95 °C lasting 3 min; 8 cycles of denaturation at 98 °C lasting 15 s, annealing at 60 °C lasting 15 s, and extension at 72 °C lasting 30 s; and ending extension at 72 °C lasting 5 s. The final cDNA library had an average insert size of 300 ± 50 base pairs. We then conducted 2 × 150 bp paired-end sequencing (PE150) using an Illumina NovaSeq™ 6000 (LC-Bio Technology, Hangzhou, China) following the manufacturer’s guidelines.

### 2.15. Bioinformatics Analysis of RNA-Sequencing (RNA-Seq)

Raw reads were processed using fastp v 0.23.2 (https://github.com/OpenGene/fastp, accessed on 30 May 2025) to remove adapter sequences, low-quality bases (Q < 20), and undetermined bases. Clean reads were aligned to the *Mus musculus* reference genome (GRCm38) using HISAT2 v 2.2.1 (https://ccb.jhu.edu/software/hisat2, accessed on 30 May 2025) with default parameters. Transcript assembly and abundance estimation were performed using StringTie v 2.1.4 (https://ccb.jhu.edu/software/stringtie, accessed on 30 May 2025), followed by transcriptome merging with gffcompare v 0.12.6 (https://github.com/gpertea/gffcompare, accessed on 30 May 2025). Gene expression levels were quantified as fragments per kilobase of transcript per million mapped reads (FPKM). The differentially expressed mRNAs were identified with fold change ≥ 2 or ≤0.5, and false discovery rate (FDR)-adjusted *p*-value < 0.05 by R package edgeR v 4.0.16 (https://bioconductor.org/packages/release/bioc/html/edgeR.html, accessed on 30 May 2025). Functional enrichment analysis of differentially expressed genes (DEGs) was performed using the Database for Annotation, Visualization and Integrated Discovery (DAVID v 6.8, https://davidbioinformatics.nih.gov, accessed on 30 May 2025). Gene Ontology (GO) terms and Kyoto Encyclopedia of Genes and Genomes (KEGG) pathways were considered significantly enriched at Q value < 0.05. Gene Set Enrichment Analysis (GSEA) was performed by GSEA v 4.1.0 (http://www.gsea-msigdb.org/gsea, accessed on 30 May 2025) with the Molecular Signatures Database (MSigDB) hallmark gene sets. Enrichment was considered significant at a nominal *p* value < 0.05 and Q value < 0.25.

### 2.16. Safety Evaluations

Mice were weighed prior to anesthesia induction with 4% isoflurane in oxygen, and blood was collected for routine blood examination on days 0, 3 and 5. PBS-treated mice were used as a negative control. Routine blood examination included white blood cell (WBC) count, neutrophil percentage (NEUT%), lymphocyte percentage (LYM%), red blood cell (RBC) count, hemoglobin concentration (HGB), hematocrit percentage (HCT%), platelet count (PLT) and plateletcrit percentage (PCT%). Major organs, including the heart, liver, lung, and kidney, were harvested at the endpoint for histological examination. H&E staining and detection were conducted as described in Section 2.13.

### 2.17. Statistical Analysis

Statistical analysis to compare vaccine formulations were performed in GraphPad Prism 10.01 (San Diego, CA, USA) and Oringin 2025 (Northampton, MA, USA). Values are presented as the means ± standard error of the mean (SEM). Significance was calculated using two-sided unpaired *t*-test and one-way ANOVA with Dunnett’s multiple comparison test or Tukey’s test when the data conformed to a normal distribution and exhibited homogeneity of variance. Otherwise, Mann–Whitney tests and Kruskal–Wallis tests were performed for nonparametric comparisons of three or more groups to the PBS group. A *p* value < 0.05 marked above the bars was considered statistically significant.

## 3. Results

### 3.1. Characterization of Microcapsule-Based Vaccines

To achieve effective vaccination, it is helpful to provide sustained antigen release of antigens in vivo [29,30,31]. A promising alternative adjuvant is a microcapsule, wherein the antigen can be loaded and shielded from degradation [11,32].

**Figure 1:**
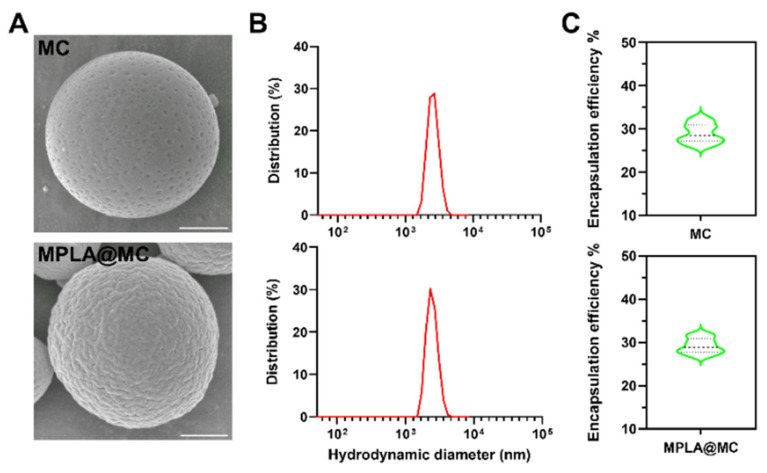
Physical characterization of uniformly sized microcapsules. (**A**) Representative SEM images of MC and MPLA@MC. Scale bars = 1 μm. (**B**) Hydrodynamic sizes of these two types of microcapsules measured by DLS. (**C**) Evaluation of encapsulation efficiency for antigen-loaded microcapsules determined by Micro BCA. The dashed lines show the median and interquartile range.

Based on our previous work [33], we prepared microsized capsules with narrow size distributions by the double emulsion method (Figure S1A). After optimization, the resulting MC and MPLA@MC exhibited a uniform spherical morphology, as revealed by SEM micrographs (Figure 1A). The hydrodynamic diameters of the two types of microcapsules were 4.58 ± 0.42 μm and 4.12 ± 0.31 μm, and the PDI was 0.20 and 0.14, respectively (Figure 1B). Additionally, their encapsulation efficiencies were up to 28.97% and 29.21%, respectively, by subtraction calculation (Figure 1C). DLS analysis further revealed that both types of microcapsules displayed negative zeta potentials, measured as −20.23 ± 0.51 mV and −21.08 ± 0.44 mV, respectively (Figure S1B).

### 3.2. Internalization, Activation, and Intracellular Trafficking

**Figure 2:**
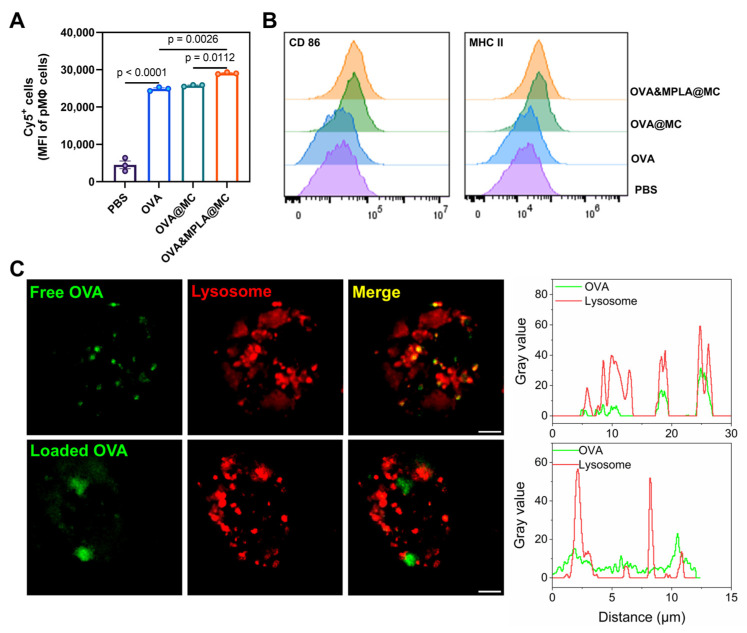
Cellular uptake of microcapsules, immune activation and intracellular trafficking. (**A**) Mean fluorescence intensity (MFI) of cellular Cy5-OVA and (**B**) flow histograms of costimulatory CD86 and MHC II molecules after 12 h incubation, detected by flow cytometry. (**C**) The colocalization of free OVA (green), OVA (green) loaded by microcapsules and LysoTracker Red DND-99 (red) after 6 h incubation, performed by CLSM and line-scan intensity profiling analyzed by ImageJ2. Fluorescence images are shown in pseudocolor. Scale bars = 2.5 μm. Data (*n* = 3) are expressed as the means ± SEMs. pMφ, peritoneal macrophage.

Antigen internalization is paramount for antigen-specific immune responses [34]. We first examined the uptake of antigens by mouse peritoneal macrophages and the cellular expression of the activation signals CD86 and MHC-II. Compared to free OVA, OVA&MPLA@MC exhibited more favorable uptake (increased by 16.95%) than OVA&MC (Figure 2A), which might be due to specific binding of MPLA to the cell surface TLR4, thereby facilitating receptor-mediated internalization. The results also showed that after incubation with different stimuli, the expression of the activation markers CD86 and MHC II was significantly upregulated in both the OVA@MC and OVA&MPLA@MC groups compared to the free OVA group (Figure 2B).

To verify the cytosolic delivery of antigen by microcapsules, we observed the intracellular distribution of OVA and colocalization with lysosomes by CLSM. Based on this observation, free OVA (green) completely colocalized with lysosomes (red), while OVA delivered by microcapsules presented less colocalization with lysosomes (Figure 2C). In a previous study of PLA microcapsules, it was confirmed that degradation product-induced acidic microenvironments likely facilitated antigen cross-presentation [18]. In our study, microcapsule-based vaccines were not confined to the conventional lysosome pathway, suggesting antigen cross-presentation.

### 3.3. Systemic Humoral Immune Responses in Serum

**Figure 3:**
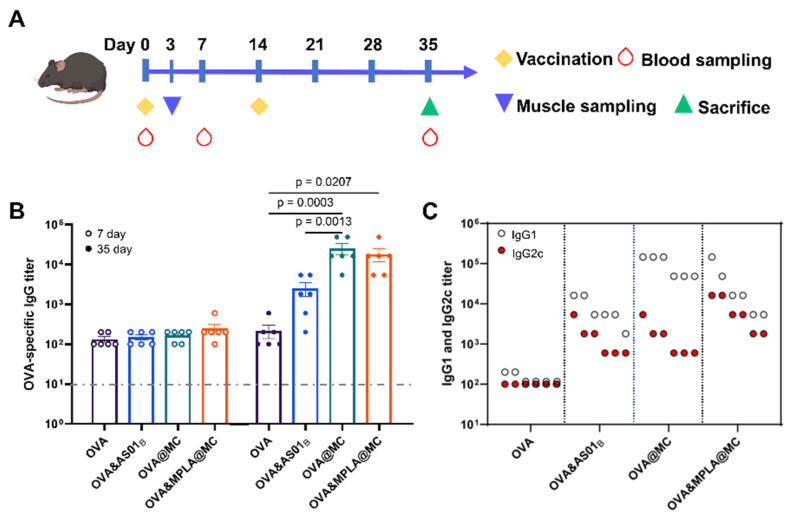
Humoral immunity evaluations of vaccines in mouse serum. (**A**) The routine immunization of formulations. (**B**) Endpoint titers of serum IgG on days 7 and 35 post-prime immunization. (**C**) Endpoint titers of serum IgG1 and IgG2c on day 35 post-prime immunization evaluated by ELISA. The titer level in the PBS group is marked by a gray dashed line. Data (*n* = 6) are expressed as the means ± SEMs. IgG, Immunoglobulin G.

To systemically evaluate the serological immune effects of different vaccine groups on days 7 and 35 (endpoint), mice were immunized with OVA, OVA&AS01_B_, OVA@MC, and OVA&MPLA@MC by intramuscular injection (Figure 3A). Antibody titers of OVA-specific IgG in all vaccine groups showed minimal differences on day 7 post-prime immunization, while the microcapsule groups all generated significantly higher IgG than the OVA&AS01_B_ group at the endpoint (Figure 3B), which might be related to the continuous supply of immunogenic OVA from the microcapsules. IgG2c indicates a Th1 immune response, whereas IgG1 is a marker of a Th2 immune response [35,36]. At the endpoint, the gap between antibody titers of IgG1 and IgG2c in OVA&MPLA@MC and OVA&AS01_B_ was minimal compared to OVA@MC, especially in OVA&MPLA@MC (Figure 3C), suggesting Th1-biased antibody production.

### 3.4. Systemic Cellular Immune Responses in Splenocytes

**Figure 4:**
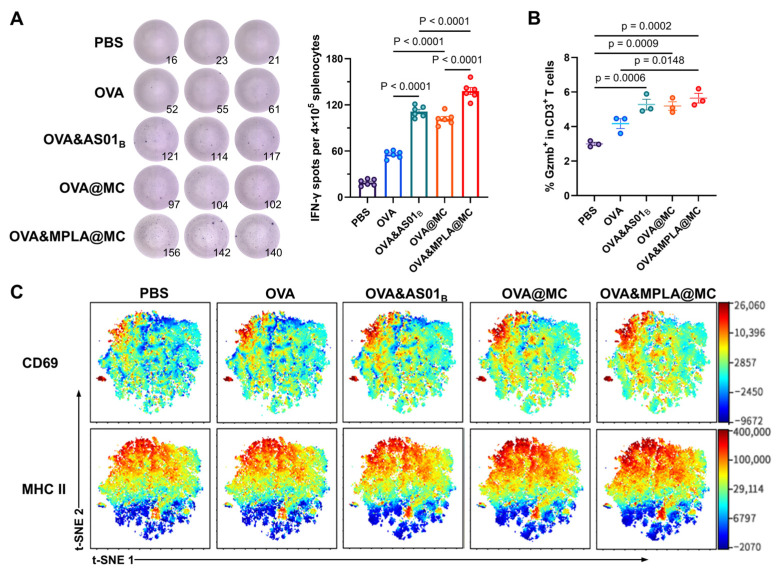
Cellular immunity evaluations of vaccines in mouse splenocytes. (**A**) Representative spots of OVA-specific IFN-γ-secreting splenocytes and quantification by ELISpot assay. (**B**) Gzmb^+^ cells among CD3^+^ T cells for characterization of cytotoxic activity measured by spectral flow cytometry. (**C**) Representative t-SNE plots showing the expression levels of CD69 and MHC II calculated by Cytobank. Color intensity indicates marker expression levels. Data (*n* = 3) are expressed as the means ± SEMs.

Encouraged by the humoral immunity outcome, we further explored the adjuvant effects of MPLA@MC on the IFN-γ secretin profile in mouse splenocytes and the activation of T cells at the endpoint. Regarding the immune tendency, OVA&MPLA@MC induced a significant increase in IFN-γ-secreting splenocytes compared to those of other groups after OVA restimulation in vitro, which was consistent with the previously confirmed Th1-biased immunity (Figure 4A). For instance, OVA&MPLA@MC exhibited up to a 24% increase in spot number compared to OVA&AS01_B_. Furthermore, OVA&MPLA@MC induced higher expression of Granzyme B and Interleukin-2 (IL-2) in CD3^+^ T cells than free OVA (Figure 4B and Figure S2A,C), indicating the successful induction of activated T cell responses and potent cytotoxic killing activity.

High-dimensional spectral flow cytometry analysis was visualized by t-SNE [37]. Comparative analysis demonstrated that OVA&MPLA@MC showed a marked shift in population distribution, characterized by increased densities of activated T cells (CD69^+^ and MHC II^+^) compared to OVA&AS01_B_ (Figure 4C). Together, Th1 immune bias as well as largely activated and multifunctional T cell activity confirmed the potent cellular immunity of OVA&MPLA@MC. 

### 3.5. Recruitment of Immune Cells to the Vaccination Site

**Figure 5:**
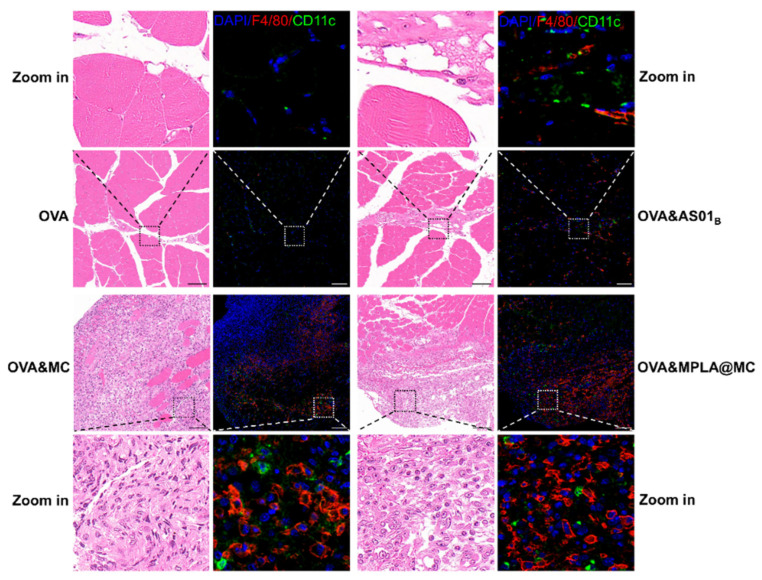
APC recruitment and distribution in muscle tissues at the vaccination site. Harvest of serial sections on day 3 postimmunization for H&E and IF analysis and representative images of local muscle tissues trapping microcapsules. H&E images were captured by optical microscopy, and IF sections were stained for nuclei (DAPI, blue), macrophages (F4/80^+^, red) and DCs (CD11c^+^, green) detected by CLSM. Zoomed images (5 × magnified) correspond to the areas indicated by the dashed boxes. Scale bars = 50 μm. DAPI, 4′,6-diamidino-2-phenylindole.

Macrophages have a powerful ability to phagocytize [38,39]. Within the optimal phagocytic range for macrophages, our microcapsules in muscle tissues at the vaccination site were observed by histopathology analysis on day 3 postimmunization. As shown in H&E images, PBS-treated tissue exhibited normal muscle architecture with intact myofibers and peripherally located nuclei (Figure S3). In contrast, microcapsule-injected tissue displayed multinucleated giant cell formation and extensive macrophage infiltration, which suggested that microcapsules underwent rapid and massive phagocytosis. Meanwhile, serial sections near the H&E section were harvested, and confocal IF images demonstrated denser infiltration of F4/80^+^ macrophages (red) in the microcapsule groups than in the OVA&AS01_B_ group, with CD11c^+^ dendritic cells (DCs, green) sparsely distributed in the surrounding areas (Figure 5). Together, rather than establishing a physical antigen depot [18], microcapsule-based vaccines operated through cellular aggregates, wherein numerous macrophages internalized and processed intracellular antigens, while DCs might rapidly migrate following antigen acquisition.

### 3.6. Transcriptomic Landscape of Vaccine-Induced Immune Activation

**Figure 6:**
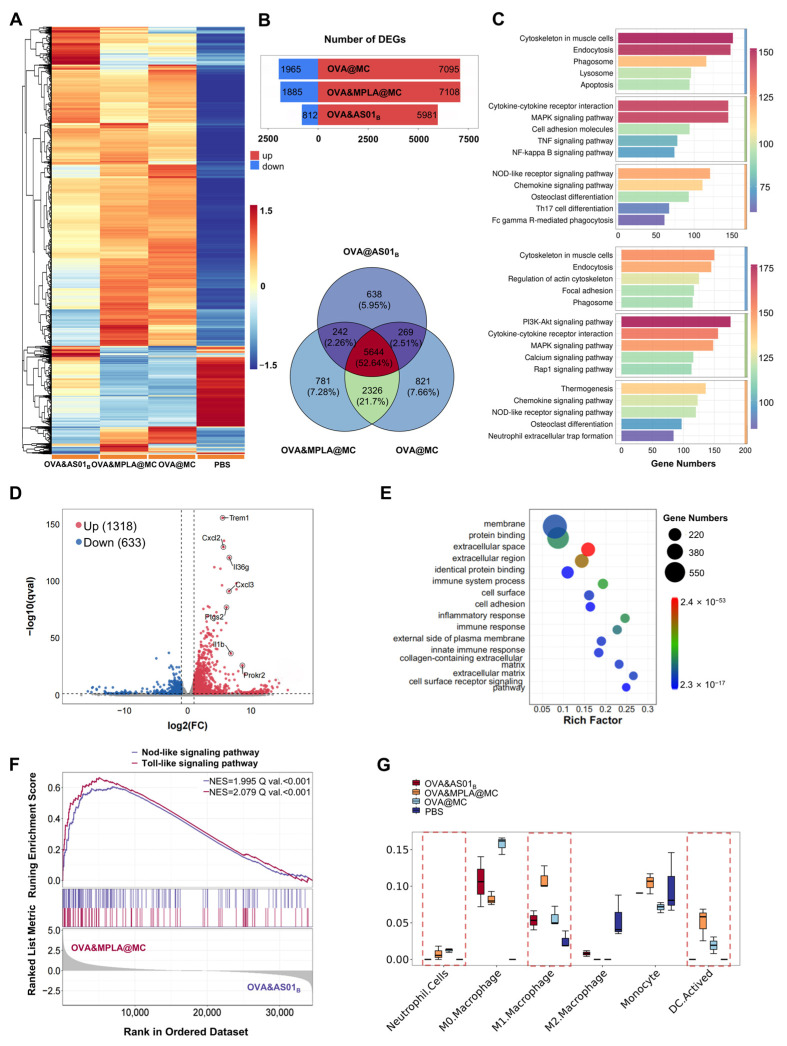
Performance of bulk RNA-seq using muscle tissues at the vaccination site. (**A**) Heatmap of DEGs (FDR-adjusted *p*-value < 0.05) in the four groups. (**B**) Numbers of DEGs in OVA&AS01_B_, OVA@MV and OVA&MPLA@MC (vs. PBS) and the overlap among correlated groups in the Venn diagram. The numbers and proportions in each sector represent the count of unique or shared DEGs. (**C**) KEGG pathway enrichment analysis of DEGs (vs. PBS) in OVA@MC (top) and OVA&MPLA@MC (bottom). Bar length indicates gene count; color scale represents Q value. (**D**) Volcano plot indicating DEGs between OVA&MPLA@MC and OVA&AS01_B_ (vs. PBS). Significant hits are depicted in red and blue (FDR-adjusted *p*-value < 0.05). Nonsignificant genes are in gray. (**E**) GO enrichment analysis of DEGs in the OVA&MPLA@MC and OVA&AS01_B_ groups (vs. PBS). Bubble size indicates gene count; color scale represents Q value. (**F**) GSEA enrichment analysis of OVA&MPLA@MC and OVA&AS01_B_ (vs. PBS) in the Toll-like and NOD-like receptor signaling pathways. (**G**) CIBERSORT analysis of the relative proportions of the indicated immune cell subsets. The dashed boxes highlight the proportions of neutrophil cells, M1 macrophages, and activated DCs. Data (*n* = 3) are expressed as the median with interquartile range. Rich Factor, ratio of enriched genes to total genes in pathway; NES, normalized enrichment score. Q val, Q value.

Healthy mice were immunized, and muscle tissues at the vaccination site were harvested on day 3 postimmunization for bulk RNA sequencing. The heatmap with hierarchical clustering of DEGs revealed distinct transcriptional profiles between different groups (Figure 6A). For analysis, OVA&AS01_B_, OVA@MC, and OVA&MPLA@MC (vs. PBS) exhibited a substantial upregulation of genes (5981, 7095, and 7108, respectively). Venn diagram analysis identified DEGs (781 and 821, respectively) exclusive to OVA@MC and OVA&MPLA@MC, suggesting distinct molecular responses (Figure 6B). Furthermore, KEGG pathway enrichment analysis of DEGs (vs. PBS) revealed significant enrichment in pathways related to the cytoskeleton in muscle cells, endocytosis, cytokine–cytokine receptor interaction, and the NOD-like receptor signaling pathway, suggesting local immune cell infiltration and activation. Notably, OVA&MPLA@MC elicited more robust innate immune responses than OVA&MC (vs. PBS), as evidenced by more enrichment genes and significance (Figure 6C).

Comparative analysis of OVA&MPLA@MC and OVA&AS01_B_ (vs. PBS) revealed substantial upregulation of DEGs (1318). Among these, marked DEGs showed high significance and were exclusively associated with participation in the transition from innate to adaptive immunity [40,41,42,43,44] (Figure 6D). GO enrichment analysis implicated active surface receptor signal transduction and extensive secretory protein release with extracellular microenvironment remodeling [45] (Figure 6E). In particular, GSEA-KEGG pathway analysis revealed significant positive enrichment of both the NOD-like and Toll-like receptor signaling pathways, indicating that OVA&MPLA@MC elicited more robust activation of innate immune sensing mechanisms in response to MPLA stimulation than AS01_B_ (Figure 6F). Finally, we employed Cell-type Identification By Estimating Relative Subsets Of RNA Transcripts (CIBERSORT) to estimate the relative abundance of immune cell subsets (Figure 6G). Consistent with previous observations, the OVA&MPLA@MC group exhibited a significantly higher relative abundance of M1-type macrophages and activated DCs than the other groups, exhibiting immunopotentiation.

### 3.7. Systemic Biosafety Profile

The evaluation of H&E staining for microcapsule-injected tissue at the endpoint exhibited attenuated nuclear aggregation and resolution of the adipose fibrotic layer, indicating reduced inflammatory cell infiltration and tissue remodeling in muscles (Figure S4A). Additionally, mice maintained a gradual weight gain over the 35-day period, with no significant differences between the vaccine groups and PBS group (Figure S4B). Hematological analysis at different time points revealed several key parameters within normal physiological ranges (Figure S4C). Furthermore, histopathological assessments of major organs (heart, liver, lung, and kidney) at the endpoint displayed normal tissue architecture in all groups, with no apparent pathological changes or inflammatory lesions (Figure S4D). Collectively, these observations confirmed the high biocompatibility of microcapsule-based vaccines.

## 4. Discussion

Achieving robust immune efficacy, especially cellular immunity, remains a major hurdle for many therapeutic and anti-infectious vaccine candidates [46,47]. High-performance platforms require sophisticated delivery systems combined with potent adjuvant activity [6]. By rationally engineering a composite adjuvant strategy, our study demonstrated that the structurally advantageous MPLA@MC exhibited spatiotemporally immune modulation and elicited synergistic immune responses.

Specifically, the integration of MPLA into the delivery system established a concerted activation profile: by gradual codegradation with microcapsules in the tissue microenvironment, MPLA provided continuous TLR4-mediated stimulation to potentiate cellular recognition and activation, synchronized with encapsulated antigen delivery through phagocyte-mediated microcapsule internalization. Such spatiotemporal coupling of microcapsule-based delivery and molecular adjuvant-triggered immunostimulation orchestrates enhanced dual immunity. Similar to the AS01_B_ adjuvant system, our vaccine incorporates MPLA. However, unlike the AS01_B_ liposomal vaccine, which is prepared by simple physical mixing, our spatiotemporally coupled MPLA@MC vaccine revealed more robust immune activation signals and a significant enrichment in Toll-like and NOD-like receptor signaling pathways in transcriptome analysis. This distinctive composite adjuvant system enabled multipronged immune signals with minimal MPLA requirements yet markedly amplified activation potency, underscoring the safety and efficacy advantages of our microcapsule-based platform.

Through novel structural engineering, our PLA-based microcapsules occupy an intermediate size regime, filling the size void between conventional nanoparticles (<500 nm) [15] and large microparticles (>10 μm) [17]. Different from large microparticles that act as static depots at the vaccination site, or nanoparticles that are prone to lysosomal degradation, these microsized capsules conferred distinct functional advantages through rapid sequestration by infiltrating myeloid cells, creating dynamic immunological aggregates, as revealed by histopathological observations. Mechanistically, we propose that these macrophages internalize microcapsules and potentially transfer antigenic cargo or processed peptides to DCs via exosomes, trogocytosis, or direct contact [48,49]. This process is instrumental in coupling innate immune activation to the subsequent initiation of adaptive immunity [50], warranting further verification in future work.

As a previous study by our research team of PLA-based gigaporous microspheres suggested [18], localized acidity may activate acid-sensing ion channels to enhance macropinocytosis-mediated antigen uptake and promote DC maturation for MHC-I processing. Notably, microcapsules exhibited reduced lysosomal colocalization, as evidenced by confocal imaging, indicating escape from conventional endosome pathways and likely promotion of cross-presentation. Accordingly, beyond enhanced humoral immunity, potent cellular immunity, characterized by an increase in IFN-γ-secreting splenocytes, Granzyme B and Interleukin-2 levels, could be observed in a series of assessments.

## 5. Conclusions

In this study, we engineered a spatiotemporally coupled delivery platform based on PLA microcapsules formulated with MPLA. The resulting OVA&MPLA@MC promoted cellular uptake and lysosomal escape in vitro, and in vivo, it induced robust humoral immunity and strong cellular immunity. Mechanistically, the microcapsules enhanced phagocyte recruitment at the injection site and activated immune-related signaling pathways, as confirmed by RNA-seq. This rationally designed composite adjuvant systems achieve an orchestrated synergy between humoral and cellular immunity, providing a safe and effective strategy for vaccination that enhances dual immune responses.

## Data Availability

The original contributions presented in this study are included in the article. Further inquiries can be directed the corresponding authors.

## Supplementary Materials

The following supporting information can be downloaded at: https://www.mdpi.com/article/10.3390/pharmaceutics18040456/s1, Figure S1: Additional physical characterization of uniformly sized microcapsules; Figure S2: The production of IL-2 in CD3^+^ T cells; Figure S3: Representative H&E and IF images of muscle tissues from the vaccination site in PBS-treated mice; Figure S4: Systemic safety evaluations.

## Abbreviations

The following abbreviations are used in this manuscript:

PLA	Polylactic acid
MPLA	Monophosphoryl lipid A
OVA	Ovalbumin
IFN-γ	Interferon-γ
Gzmb	Granzyme B
CMI	Cell-mediated immune
TLR	Toll-like receptor
SEM	Emission scanning electron microscopy
PDI	Polydispersity index
DLS	Dynamic light scattering
ELISA	Enzyme-linked Immunosorbent Assay
ELISpot	IFN-γ Enzyme-linked Immunospot
t-SNE	t-distributed Stochastic Neighbor embedding
MC	Microcapsules
DEGs	Differentially expressed genes
GO	Gene Ontology
KEGG	Kyoto Encyclopedia of Genes and Genomes
GSEA	Gene Set Enrichment Analysis
IL-2	Interleukin-2
DCs	Dendritic cells
CIBERSORT	Cell-type Identification by Estimating Relative Subsets of RNA Transcripts
