# Supplementary material for: Engineered Polylactic Acid (PLA) Microcapsules for Spatiotemporally Coupled Delivery and Synergistically Enhanced Dual Immunity
Source: Pharmaceutics. 2026 Apr 9;18(4):456. doi: 10.3390/pharmaceutics18040456 (PMC13118379; doi:10.3390/pharmaceutics18040456)

Supplementary Figure S1. Additional physical characterization of uniformly sized microcapsules. (**A**) Representative wide-field SEM images of microcapsules. Scale bars = 1.5 μm. (**B**) Zeta potential of MC and MPLA@MC. Data (n = 6) are expressed as the means ± SEMs.

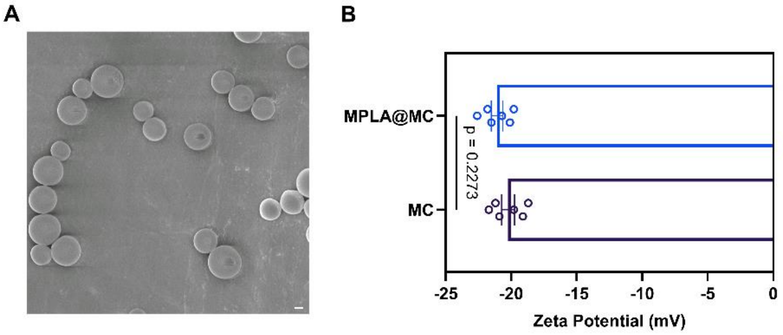

Supplementary Figure S2. The production of IL-2 in CD3+ T cells. (**A**) IL-2+ cells among CD3+ T cells for characterization of polyfunctional T cells. Data (n = 3) are expressed as means ± SEMs. (**B**) The gating strategy employed to identify T cells. (**C**) Representative flow cytometry plots showing the expression of IL-2 in CD3+ T cells corresponding to the groups in (**A**).

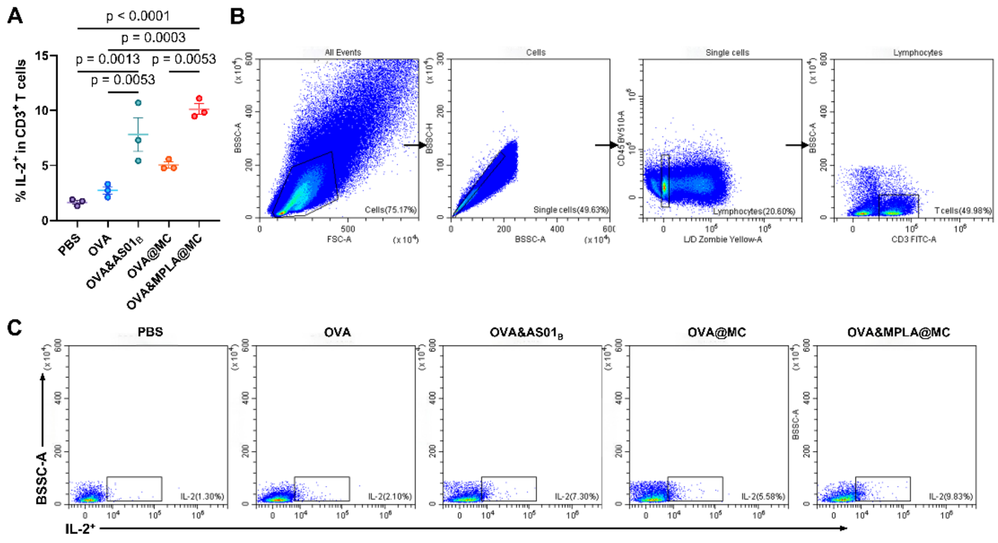

Supplementary Figure S3. Representative H&E and IF images of muscle tissues from the vaccination site in PBS-treated mice. IF sections stained for nuclei (DAPI, blue), macrophages (F4/80$^+$, red) and DCs (CD11c$^+$, green) detected by CLSM. Zoomed images (5× magnified) correspond to the areas indicated by the dashed boxes. Scale bars = 50 μm.

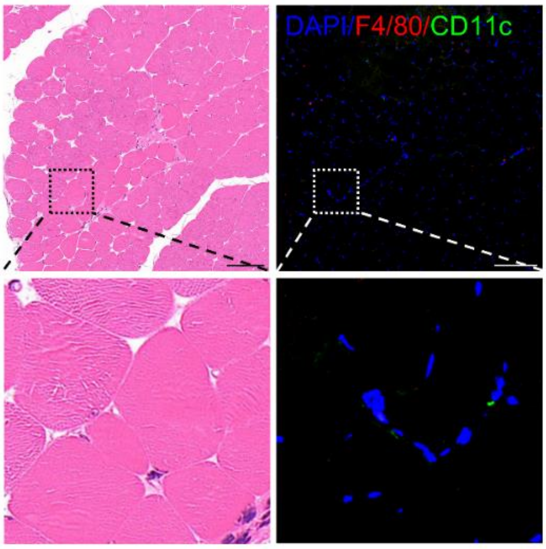

Supplementary Figure S4. Systemic safety evaluations. (**A**) Representative H&E images of muscle tissues from the vaccination site in microcapsule-treated mice. Scale bar = 50 μm. (**B**) Measurement of body weight on days 0, 3 and 35 post prime-immunization. (**C**) Hematological profiles on days 0, 3, 5 and 35 post prime-immunization. Normal range is demarcated by a gray dashed lines. (**D**) histological profiles of major organs (heart, liver, lung, and kidney) at the endpoint. Scale bar = 100 μm.

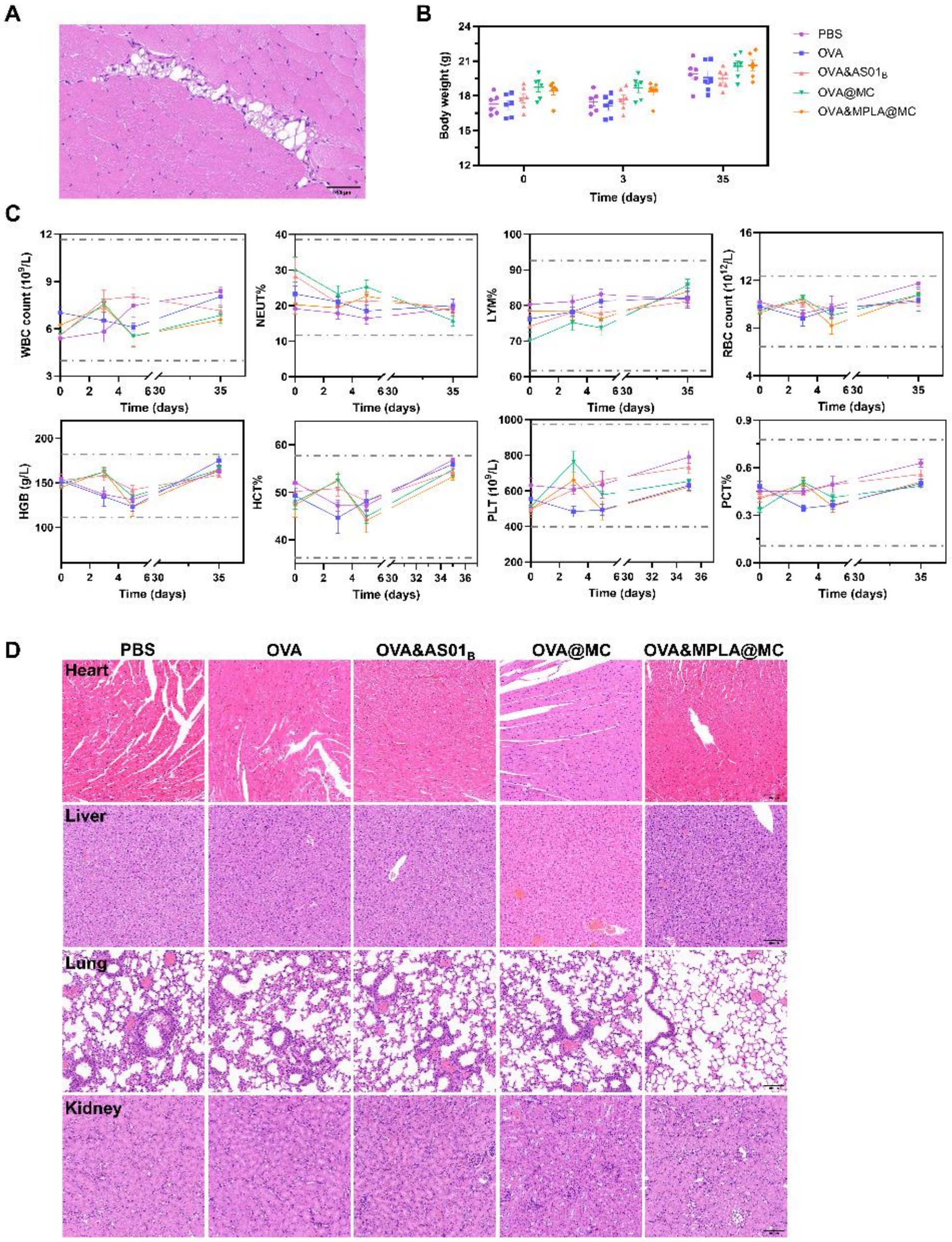

Supplement: Supplementary file 1 [file pharmaceutics-18-00456-s001.zip › pharmaceutics-4207204-supplementary.pdf]
